# Retrospective Study Comparing Clinical Outcomes of Fixed Dental Prostheses in Matched Groups of Bruxer and Nonbruxer Patients

**DOI:** 10.1155/2022/6818170

**Published:** 2022-03-27

**Authors:** Mohammed Hawthan, Bruno R. Chrcanovic, Christel Larsson

**Affiliations:** ^1^Department of Prosthodontics, Faculty of Odontology, Malmö University, Malmö, Sweden; ^2^Department of Oral and Maxillofacial Surgery and Diagnostic Sciences, Faculty of Dentistry, Umm Al-Qura University, Makkah, Saudi Arabia

## Abstract

**Background:**

Tooth-supported fixed dental prosthesis (FDP) is one of the most reliable treatment options to replace missing teeth. The longevity of the treatment could, however, be affected by several general and local factors, especially bruxism.

**Objective:**

To investigate the influence of bruxism on the long-term survival of tooth-supported FDPs in bruxers compared to a matched group of nonbruxers, taking several clinical variables into account.

**Materials and Methods:**

The present retrospective cohort study was based on records of patients treated with 3–7-unit tooth-supported FDPs with a minimum follow-up time of 6 months after prosthesis delivery. The criteria for the diagnosis of “possible” and “probable” sleep or awake bruxism were used. A matched group of nonbruxers was selected on the basis of similarities in four factors, patients' gender and age, number of prosthetic units of the FDPs, and follow-up time. The paired-samples *t*-test or Wilcoxon signed rank test were used to compared mean values between the two groups. Contingency tables of categorical data were analyzed by McNemar's test.

**Results:**

The cohort group consisted of 62 noncantilevered FDPs in each group, followed up for a mean of 110.1 and 106.5 months (bruxers and nonbruxers, respectively). Tooth-supported FDPs in bruxers presented significantly higher failure rate than in nonbruxers (32.3% vs. 25.8%, respectively; *p* = 0.001). Loss of retention and tooth loss were the main reasons for failures in both groups. For nonsmokers, the FDP failure rate was higher in nonbruxers. Technical and biological complications were significantly more prevalent in bruxers compared to nonbruxers.

**Conclusions:**

Bruxism is suggested to increase technical and biological complications and FDP failure.

## 1. Background

Tooth-supported fixed dental prostheses (FDPs) are used to replace missing tooth substance and missing teeth in partially edentulous patients [1] to restore oral function and aesthetics [2]. FDPs have generally shown high survival rates [2–7], but complications occur, and one of the main suggested factors to affect survival and complication rates is bruxism [8–11].

Bruxism is defined as a repetitive jaw-muscle activity represented by clenching and grinding of the teeth and/or by bracing or thrusting of the mandible during wakefulness and sleep [12]. The etiology of bruxism is not fully clear [13]. Several factors have been reported as risk factors for bruxism including young age, female gender, tobacco usage, alcohol drinking, caffeine consumption, stress, anxiety, obstructive sleep apnea, genetic, and certain medications [14–16].

The prevalence of bruxism has been estimated at around 10% of the population, but some studies suggest a prevalence of up to a third of the population [13, 17]. The wide variations in prevalence and the lack of accurate estimation among studies is due to different methods applied to diagnose bruxism and different selected samples [14, 17]. Since the estimated number of people with bruxism is considerable, it is important to study the influence of bruxism on the survival of FDPs.

Technical complications such as loss of retention and material fracture have been reported to occur more frequently in bruxers compared to nonbruxers [10, 14]. The risk of metal-ceramic defect of FDPs has been reported to be 2.5 times higher in bruxers compared to nonbruxers [11].

Several studies have reported a possible association between bruxism and technical complications of FDPs, but, as far as we know, there is still limited evidence concerning the survival of FDPs when subjected to multiple factors in a clinical setting and few studies compare matched groups of bruxer and nonbruxer patients [8, 10, 11]. Investigating the outcomes under the influence of many clinical independent variables, better represent a real-life situation. Recognizing conditions that place the patient at high risk of failure will allow the clinician to make an appropriate decision and refine the treatment plan to optimize the treatment outcome.

The aim of the present retrospective study was to evaluate and compare the failure rates and technical and biological complications of FDPs in a group of patients presenting bruxism in comparison with a matched group of nonbruxers with respect to several clinical variables. The null hypothesis was that there is no influence of bruxism on the survival of tooth-supported FDPs.

## 2. Materials and Methods

### 2.1. Study Design

This retrospective study included patients rehabilitated with tooth-supported FDPs at the Faculty of Odontology, Malmö University, Sweden, during the period between 1981 and 2018. The prosthetic treatments were executed by dental students, general practitioners, or specialists at a prosthodontic and general adult dental care department. The study protocol was approved by the Regional Ethical Committee in Lund, Sweden (Dnr 4,3-2018/422; Dnr 2018/856). This observational study followed the STROBE guidelines [18].

### 2.2. Definitions

Success was defined as a prosthesis that had remained unchanged (no complication or intervention) over the observation period. Survival was defined as the prosthesis remaining in situ with the occurrence of any complication while still in function. A prosthesis removed or replaced was considered a failed prosthesis. Chipping was defined as loss of veneer substance with or without metal exposure, being classified as minor (managed chair-side) or major (sent to the dental lab for reparation). An abutment was considered as a failure if exhibiting extensive alveolar bone loss and/or excessive tooth mobility, extensive caries, or any other complication that would make the abutment unsuitable to function as a retainer for FDP and lead to abutment loss.

The criteria for the diagnosis of “possible” and “probable” sleep or awake bruxism were used. A “possible” sleep or awake bruxism should be based on self-report using questionnaires and/or the anamnestic part of a clinical examination. “Probable” bruxism should be based on self-report together with clinical examination [12]. The information from the self-report and clinical examination was verified from annotations on the dental records of each eligible patient.

### 2.3. Inclusion and Exclusion Criteria

The inclusion criteria consisted of patients treated with tooth-supported FDPs (limited to 3–7 prosthetic units) at the aforementioned faculty, who had an available dental record at the clinic's archive, and a minimum follow-up time of 6 months after delivery of the prosthetic work. The exclusion criteria consisted of cases with lack of information regarding the outcome measures in the dental records, FDPs smaller or larger than 3–7 units, single crowns, and cantilever bridges.

### 2.4. Data Collection

The patients' data were collected and inserted directly into a database in a SPSS file (SPSS software, version 27, SPSS Inc., Chicago, USA) by one investigator. The following data were collected: patient's age at the day of prosthesis delivery, sex, abutment vitality, type of prosthesis material, number of prosthetic units, position of the nonvital abutment(s), presence of post and core, area of placement (anterior, posterior, and anterior/posterior), location of the prostheses in relation to the jaws (maxilla, mandible), smoking habits, bruxism, and complications after delivery of the definitive prosthetic restoration including the date when they occurred. Types of materials used in FDPs were registered according to the dental lab order. Technical and biological complications were evaluated radiographically and through the patients' records. Biological complications included caries, loss of tooth vitality, periapical infection, mobility, and abutment loss. Technical complications included tooth fracture, loss of retention, framework fracture, and minor and major veneer chipping.

### 2.5. Formation of a Matched Group

From the group of patients not presenting bruxism, a control group was included with the same number of patients as in the study group. The matching was performed in SPSS (version 27 software, IBM Inc., Chicago, IL, USA). The matches were selected on the basis of similarities in four factors, namely, patients' gender (equal matching), age of the patients at the day of prosthesis delivery (tolerance of 10 years of difference), number of prosthetic units of the FDPs (equal matching), and follow-up time (tolerance of 18 months of difference).

### 2.6. Statistical Analysis

The mean, standard deviation (SD), and percentage were calculated for several variables. The Kolmogorov–Smirnov test was performed to evaluate the normal distribution of the variables, and Levene's test evaluated homoscedasticity. The performed tests for the comparison of mean values between the two groups were the paired-samples *t*-test or Wilcoxon signed rank test, depending on the normality, and McNemar's test was used in the analysis of contingency tables of categorical data. The degree of statistical significance was considered *p* < 0.05. All data were statistically analyzed using SPSS. There was only one prosthesis per patient. Therefore, there was no clustering effect.

## 3. Results

The cohort group consisted of 331 noncantilevered FDPs with 3–7 prosthetic units followed up for a minimum of 6 months, of which 102 were installed in “possible” or “probable” bruxers. The matching according to the tolerance set for the four aforementioned factors was possible for 62 of these 102 FDPs in bruxers, which were matched to 62 FDPs in nonbruxers.

Table 1 provides the descriptive characteristics of the matched groups. As expected from the matching process, the groups did not significantly differ with regard to number of FDPs, the number of FDPs installed in patients of different sexes, the number of FDPs with the respective number of prosthetic units, the mean age of the patients at the day of prosthesis delivery, and the follow-up time. The number of prostheses in smokers did not differ between the two groups.

FDPs in bruxers presented significantly higher failure rates of 32.3% (*p* < 0.001) in comparison to nonbruxers 25.8%. Failure rates were higher in women, in the maxilla, in the posterior region of the jaws, when all abutment teeth of the prosthesis were vital, for prostheses fabricated with gold-based metal-ceramic (Au-MC), and when the treatment was conducted by dental students. Loss of retention and tooth loss were the main reasons for FDPs failures in bruxers and nonbruxers (Table 2). For nonsmokers, the FDP failure rate was higher in nonbruxers in comparison to bruxers (Table 1).

Technical and biological complications were significantly more prevalent (*p* < 0.001) in bruxers than in nonbruxers (complication level), although not when only the number of prostheses with at least one complication (prosthesis level) was compared between the groups (Table 1).

Cross-tabulation was performed with three variables (bruxism, smoking, and treatment provider) in order to verify whether there was a possible association with treatment provider to the higher prosthesis failure rate in nonsmokers among nonbruxers than among bruxers. Cross-tabulation showed that all prosthetic treatments for nonsmoker and nonbruxer patients were conducted by dental students, whereas all types of treatment provider were represented in the other groups (Table 3).

## 4. Discussion

In the present study, a higher failure rate was observed among FDPs in bruxers compared to FDPs in the matched group of nonbruxers. The same was observed for the prevalence of technical and biological complications. The influence of bruxism on the survival of FDPs was also suggested by previous studies, with a high prevalence of technical complications [10, 11].

Loss of retention and tooth loss were the main reasons for FDPs failures in both groups. Both complications occurred more commonly in bruxers compared to nonbruxers. The findings are in agreement with previous studies where loss of retention has been reported to occur more frequently in bruxers [10, 11]. Excessive overload could induce micromotion of the framework and may cause loss of retention [10]. This event is dependent on the fracture resistance of the luting cement filling the irregularity between fitting surface of FDP and abutment [9]. Loss of retention could also occur between FDP and abutment or between post and core and abutment tooth [9]. Furthermore, loss of retention could relate to the minimum resistance and retention form of the worn dentition in bruxers [14]. Subgingival preparation to increase the amount of tooth structure and create space for the FDP materials may be required [14]. Abutment tooth loss could also be a consequence of the excessive loading force by bruxers. In periodontitis patients, overloading force could enhance periodontal breakdown and negatively influence the alveolar bone density and create a bony defect which leads to pathological tooth mobility and may cause tooth loss [19–21].

In the present matched groups, major porcelain chipping occurred only in bruxers. Porcelain chipping was also reported as one of the main technical complications in bruxers [10, 11]. The excessive loading by bruxers could increase the susceptibility of porcelain chipping, initiated with roughness of the porcelain veneer at the occlusal surface which would propagate until chipping occurs [14, 22]. Fabricating FDPs with only metal at the occlusal surface might help to improve the treatment prognosis and eliminate the occlusal chipping complication [23]. The risk of porcelain chipping for the posterior FDPs could be higher compared to the anterior ones, since the posterior teeth are subjected to higher masticatory forces [24–26].

Recurrent caries was the most common complication in bruxers. Recurrent marginal caries could be a consequence of loss of retention of FDPs [9]. It is difficult to precisely predict which one occurred first: the process could start with loss of retention followed by caries or the opposite way if there is an open margin [9]. However, as a clinical examination was not performed, the precise primary cause cannot be ascertained. Marginal discrepancies, as open and under extended margins, are also among the factors that may have an influence on loss of retention and recurrent caries [27]. The uncovered prepared rough tooth structure can easily retain the plaque and increase the susceptibility of marginal caries [27]. Karlsson found a significantly higher incidence of marginal caries in open margin and poor adaptation FDPs compared to FDPs with no open and underextended margins [27]. Regular follow-up involving FDPs and surrounding structure is an important factor to achieving the long-term prognosis [27].

Loss of tooth vitality was observed to be one of the main biological complications for both groups. Mechanical trauma during tooth preparation with the insufficient cooling system and little remaining dentin thickness may increase the risk of pulpal irritation and lead to pulp necrosis [28, 29]. Detailed investigation for the loss of tooth vitality was not possible due to the record-based retrospective design of the study.

A higher failure among vital abutments is an unexpected finding as it is generally a risk reducing factor compared to nonvital abutments. In a previous study, lower survival rates were reported for FDPs with at least one nonvital abutment compared to FDPs supported by vital ones [30]. Excessive removal of the dentin structure by mechanical and chemical debridement of the root canal system may compromise the tooth structure and reduced the fracture resistance [31, 32]. Furthermore, increasing the brittleness of the dentin structure of the nonvital abutments may increase the incidence of the tooth fracture [29]. A possible reason for the higher failure rate of FDPs with vital abutments in bruxers could be due to status of the dentition and opposing arch which may have an influence on the magnitude of the bite force among bruxers and could thus affect the prognosis of FDPs [33, 34]. Any influence of the status of the opposing teeth could, however, not be evaluated in the present study. The status of the opposing arch could have changed from baseline to subsequent follow-ups without mention in patients' records, and it is thus difficult to judge whether a complication happened when FDP was opposed by natural dentition or when the opposing arch received fixed or removable prosthesis.

FDPs made of Au-MC material had a higher failure rate in bruxers. Possible explanations for these results could be the area of placement factor. Most of FDPs made of Au-MC material were delivered in the posterior region of the bruxer patients which is subjected to higher masticatory force compared to anterior region [24–26, 35].

There was a higher failure rate of FDPs for bruxers in the maxilla and in women in comparison to nonbruxers. Some factors were not matched between groups, e.g., treatment provider, status of the dentition, and opposing arch. These factors could have had an influence on the results. Treatment results obtained by dental students are not expected to be equal to the treatment results obtained by general practitioners and specialists [36]. The failure rates of FDPs for nonsmoker patients were higher in nonbruxers compared to bruxers. The reason could relate to the treatment provider factor, as all prosthetic treatments for nonsmoker and nonbruxer patients were conducted by dental students only.

There are limitations with record-based retrospective studies. The clinical procedures were not standardized as it could be in a prospective study, and the professionals involved in the treatment of these patients were not calibrated. Moreover, some data may not have been completely recorded in the patients' journals at each follow-up appointment, which is connected to retrospective nature of the present study. The lower sample size of subgroups could also be considered as one of the limitations of the present study. Clinical diagnosis of bruxism is difficult, so the present group of patients could be either under or overdiagnosed [8, 37]. Misclassification and underdiagnosis of bruxism could affect the treatment prognosis. Self-reporting questionnaire combined with clinical examination could be a practical and an appropriate way for the diagnosis of bruxism [12, 38]. Furthermore, well-designed randomized clinical trials (RCTs) studies are needed to assess the risk of bruxism on the survival of FDPs.

Nevertheless, identifying bruxer patients before prosthetic treatment might help the clinician highlight risks during planning and execution in order to reduce technical and biological complications and improve treatment outcome [11].

## 5. Conclusion

Within the limitations of this retrospective study, bruxism is suggested to contribute to FDP failure due to an increased prevalence of technical and biological complications.

## Figures and Tables

**Table 1:** Descriptive characteristics of the matched groups and comparison of the prosthesis failure rates between bruxers and nonbruxers.

	Bruxers	Nonbruxers	*P* value
FDP (*N*)	62	62	
Sex (number of FDP)	Woman (44)	Woman (44)	
	Man (18)	Man (18)	
Prosthetic units (number of FDP)	3 (40), 4 (16), 5 (4), 6 (2)	3 (40), 4 (16), 5 (4), 6 (2)	
Patients' age^†^ (years), mean ± SD (min-max)	57.8 ± 11.7 (31.9–81.5)	58.3 ± 11.1 (37.7–84.3)	0.729^††^
Follow-up (months), mean ± SD (min-max)	110.1 ± 66.6 (15.3–262.1)	106.5 ± 66.7 (6.0–259.3)	0.767^‡‡^
Smoking (number of FDP)^‡^			0.743^§§^
** **No	20	13	
** **Current + former smokers	15	17	
FDP failure, *N* (%)	20 (32.3)	16 (25.8)	0.001^§§^
** **Sex, *N*/total (%)			
** **Woman	12/44 (27.3)	10/44 (22.7)	0.002^§§^
** **Man	8/18 (44.4)	6/18 (33.3)	0.503^§§^
** **Smoking, *N*/total (%)^‡^			
** **No	5/20 (25.0)	4/13 (30.8)	0.019^§§^
** **Current + former smokers	6/15 (40.0)	7/17 (41.2)	0.804^§§^
** **Prosthesis location, *N*/total (%)			
** **Maxilla	12/37 (32.4)	6/39 (15.4)	0.002^§§^
** **Mandible	8/25 (32.0)	10/23 (43.5)	0.383^§§^
** **Area of placement, *N*/total (%)			
** **Anterior	3/8 (37.5)	1/8 (12.5)	0.219^§§^
** **Posterior	15/38 (39.5)	10/31 (32.3)	0.035^§§^
** **Anterior/posterior	2/16 (12.5)	5/23 (21.7)	0.064^§§^
** **Abutments, *N*/total (%)			
** **All abutments vital	6/31 (19.4)	6/41 (14.6)	0.001^§§^
** **Vital and nonvital abutments	13/27 (48.1)	10/20 (50.0)	0.541^§§^
** **All abutments nonvital	1/4 (25.0)	0/1 (0)	0.250^§§^
** **FDPs with post and core in nonvital abutments, *N*/total (%)^§^			
** **No	3/12 (25.0)	6/11 (54.5)	0.727^§§^
** **Yes	11/19 (57.9)	4/10 (40.0)	0.332^§§^
** **Position nonvital abutment, *N*/total (%)			
** **Mesial	9/14 (64.3)	2/6 (33.3)	0.267^§§^
** **Distal	2/10 (20.0)	7/12 (58.3)	0.453^§§^
** **Mesial and distal	2/5 (40.0)	0/2 (0)	1.000^§§^
** **Middle	1/2 (50.0)	1/1 (100)	1.000^§§^
** **Material, *N*/total (%)^¶^			
** **Gold ceramic	16/38 (42.1)	7/38 (18.4)	0.040^§§^
** **CoCr ceramic	4/16 (25.0)	8/18 (44.4)	0.180^§§^
** **Treatment provider, *N*/total (%)			
** **Dental student	15/45 (33.3)	10/51 (19.6)	0.001^§§^
** **General or specialist dentist	5/17 (29.4)	6/11 (54.5)	1.000^§§^
** **FDPs with at least one complication (*N*)	27	22	0.111^§§^
Complications (*N*)			
** **Technical			<0.001^§§^
** **Major chipping	3	0	
** **Loss of retention	8	6	
** **Prosthesis replacement	7	3	
** **Tooth fracture	2	1	
** **Biological			<0.001^§§^
** **Loss of tooth vitality	7	8	
** **Recurrent caries	12	7	
** **Periapical destruction	2	1	
** **Technical/biological			<0.001^§§^
** **Tooth loss	8	5	

FDP, fixed dental prosthesis. ^†^On the day of prosthesis delivery. ^‡^Information not available for all patients. ^§^Only for the prostheses that had at least one nonvital abutment tooth. ^¶^Analysis done only for the most prevalent materials used. ^††^Wilcoxon signed rank test. ^‡‡^Paired-samples *t*-test. ^§§^McNemar's test.

**Table 2:** The reasons for FDPs failures in bruxers and nonbruxers.

Reasons for FDPs failures	Bruxers		Nonbruxers	
	Frequency	Percentage	Frequency	Percentage
Loss of retention	5	25.0	4	25.0
Tooth loss	5	25.0	4	25.0
Tooth fracture (root or crown)	2	10.0	1	6.3
Major chipping	2	10.0	0	0
Periapical destruction (radiolucency)	2	10.0	1	6.3
Replace old construction by new one due to change situation	2	10.0	1	6.3
Loss of tooth vitality	1	5.0	2	12.5
Mobility	1	5.0	0	0
Esthetic dysfunction	0	0	1	6.3
Allergy	0	0	1	6.3
Total	20	100.0	16	100.0

FDPs, fixed dental prostheses.

**Table 3:** Cross-tabulation of smoking, history of bruxism, and treatment provider factors to verify a possible association of treatment provider to the higher prosthesis failure rate in nonsmokers among nonbruxers.

Treatment provider			History of bruxism		Total
			No	Yes	
Dental student	Smoking	No	13	12	25
		Yes	9	7	16
		Quit smoking	2	5	7
	Total		24	24	48
General practitioner or specialist	Smoking	No	0	8	8
		Yes	6	2	8
		Quit smoking	0	1	1
	Total		6	11	17
Total	Smoking	No	13	20	33
		Yes	15	9	24
		Quit smoking	2	6	8
	Total		30	35	65

## Data Availability

The data used to support the findings of this study are obtained from patients treated at the XXXXXX, and cannot be shared, in accordance with the General Data Protection Regulation (EU) 2016/679 and are restricted by the Regional Ethical Committee in Lund, Sweden (Dnr 4,3-2018/422; Dnr 2018/856), in order to protect patient privacy.
